# Tympanic Resonance Hypothesis

**DOI:** 10.3389/fneur.2020.00014

**Published:** 2020-01-30

**Authors:** Michael J. O. Boedts

**Affiliations:** ^1^Brai3n, Ghent, Belgium; ^2^ENT Department, AZ Maria Middelares, Ghent, Belgium

**Keywords:** ear diseases, tympanic membrane, attention, tinnitus, hyperacusis, Eustachian tube, auditory perception, trigeminal nuclei

## Abstract

Seemingly unrelated symptoms in the head and neck region are eliminated when a patch is applied on specific locations on the Tympanic Membrane. Clinically, two distinct patient populations can be distinguished; cervical and masticatory muscle tensions are involved, and mental moods of anxiety or need. Clinical observations lead to the hypothesis of a “Tympanic Resonance Regulating System.” Its controller, the Trigeminocervical complex, integrates external auditory, somatosensory, and central impulses. It modulates auditory attention, and directs it toward unpredictable external or expected domestic and internal sounds: peripherally by shifting the resonance frequencies of the Tympanic Membrane; centrally by influencing the throughput of auditory information to the neural attention networks that toggle between scanning and focusing; and thus altering the perception of auditory information. The hypothesis leads to the assumption that the Trigeminocervical complex is composed of a dorsal component, and a ventral one which may overlap with the concept of “Trigeminovagal complex.” “Tympanic Dissonance” results in a host of local and distant symptoms, most of which can be attributed to activation of the Trigeminocervical complex. Diagnostic and therapeutic measures for this “Tympanic Dissonance Syndrome” are suggested.

## Introduction

**Figure 1:**
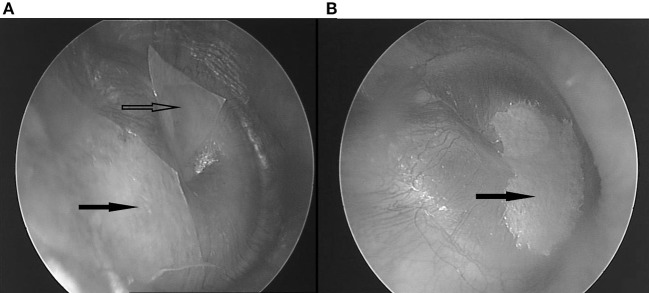
**(A)** Two patches covering the superior half. Hollow arrow: anterosuperior patch; black arrow: posterosuperior patch. **(B)** One bigger patch, covering the inferior half. Black arrow: inferior patch.

The etiology of many complaints in and around the ear is poorly understood. It was found that some respond to application of paper patches on the tympanic membrane (TM): autophony (1, 2) (a disturbing echo-like perception of one's own voice) and fullness feeling in the ear (3). Often, these patients show accompanying symptoms (4), that sometimes respond to patching as well (1, 3): pulsating sounds, clicks, or rhythmic sounds in the ear, hyperacusis, tension type headache, feeling of slime in the throat, lump feeling, certain equilibrium problems, burning mouth syndrome, … It appears that these often respond without autophony or fullness feeling, or even ear complaints, being present. Clinically, two discernable patient groups emerge: a “dorsal” one, in which the symptom cluster partly overlaps with the one described in Tensor Tympani syndrome (5), in which patients show tender point in the dorsal cervical muscles and masticatory muscles, and complaints respond to patching of the upper half of the TM; and a “ventral” group, in which the symptom cluster is remarkably similar to the one of “sensory laryngeal neuropathy” (6), in which pain is elicited by palpation of the prevertebral muscles and/or the trigger-point of the superior laryngeal nerve (the location just lateral from cornu majus of the hyoid), and symptoms respond to patching of the lower half of the TM (Figure 1).

Dull hearing may become *clearer* or *brighter* after patching. Many patients suffer from anxiety or stress; some report earlier complaints as oppressive feeling on the chest, palpitations, gastro-intestinal complaints; have had sciatica or carpal tunnel surgery; neuropathic symptoms, or are diagnosed with fibromyalgia or chronic fatigue syndrome. In most cases no distinct etiology can be found.

These clinical observations have led to formulating the present hypothesis. As yet, only limited clinical data on patching have been published, and many statements in this hypothesis are derived from unpublished clinical experience with tympanic patching.

### Anatomy

**Figure 2:**
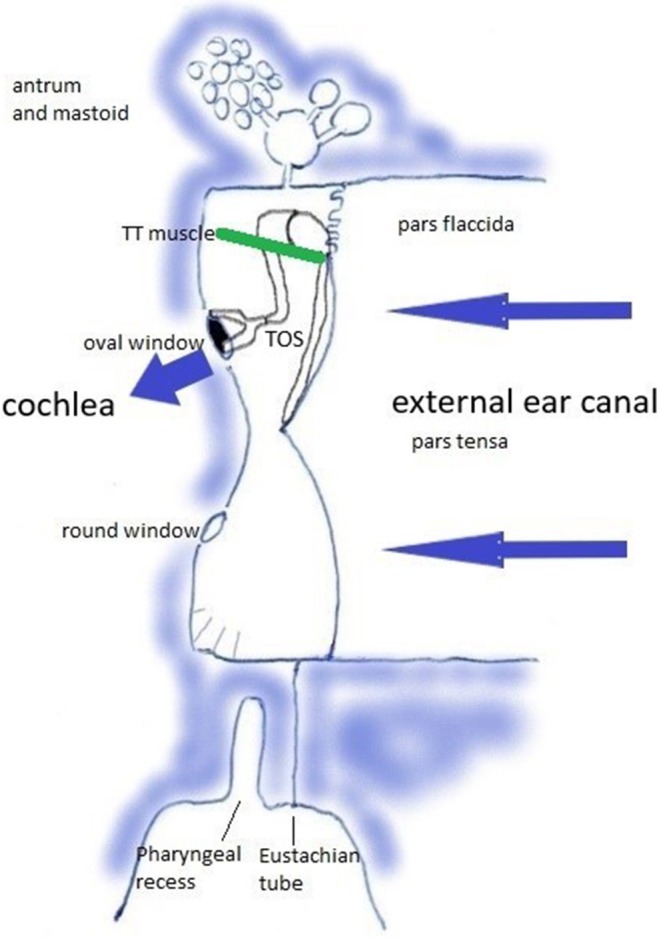
The Tympano-Ossicular System. Blue arrows: sound wave transmission. Green line: tensor tympani muscle.

Relevant anatomy can be studied in https://en.wikipedia.org/wiki/Tympanic_cavity and https://en.wikipedia.org/wiki/Pharyngeal_recess. The middle ear transmits sound waves from the air toward the cochlea. This transfer function rests on the tympano-ossicular system (TOS) (Figure 2), which consists of the three compliant elements in the capsule [the oval window (OW), round window (RW), and TM], and the ossicles with attached muscles [of which the tensor tympani muscle (TT) plays an central role in this hypothesis]. TOS stiffness is defined by the stiffness of the TM, of the suspending ligaments and ossicular joints, the OW annular ligament, the RW; and by the degree of muscular contraction. It further depends on cochlear loading, which is related to intracochlear pressure and cerebrospinal fluid pressure, and cochlear anatomy integrity. In this sound transfer system, two regions are subject to modification by muscular contraction: the TM, and the pharyngeal part of the Eustachian tube (ET) with the adjacent Pharyngeal Recess (PR).

**Figure 3:**
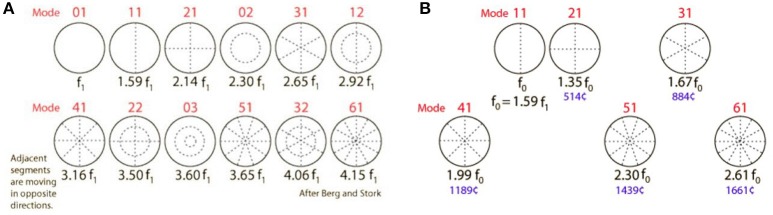
**(A)** Modes of an ideal circular membrane. When the air is not compressed in a cavity, the membrane vibrates freely in the (0,1) mode, which does not produce a pleasing tone and is detrimental to pitch and sound quality (see also: https://www.acs.psu.edu/drussell/demos/membranecircle/circle.html). Note the non-harmonicity when all modes are excited. **(B)** In the music instrument called “kettle drum” or “timpani,” compression of the air in the bowl results in damping of the unwanted and disturbing fundamental (0,1) mode; and correct tempering results in quasi-alignment of the preferred modes, so that a quasi-harmonic series of overtones is formed. This series defines pitch, harmonicity, timbre, and clarity of the sound. The middle ear cavity exerts a similar effect on TM resonance frequencies. R. Nave, with permission: https://www.mwit.ac.th/~physicslab/hbase/music/cirmem.html.

**Figure 4:**
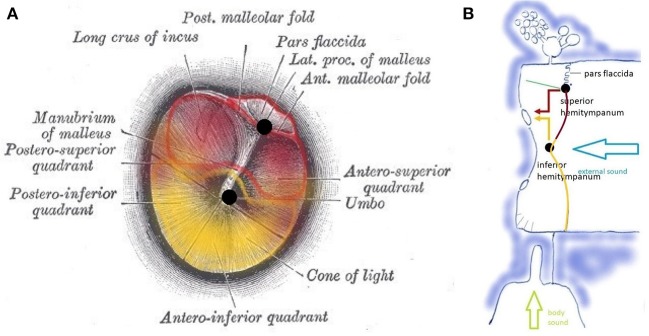
**(A)** Tympanic membrane with a pars flaccida and pars tensa. Functionally, the pars tensa contains two separate entities or “hemitympani” which roughly coincide with the upper and lower half of the TM. Upper hemitympanum: red; lower hemitympanum: yellow; (from Gray's anatomy via Wikipedia). **(B)** Vibrations of the upper hemitympanum, related to lower frequencies (red arrow) are mainly transmitted via the superior tympano-mallear connection (upper black dot), those of the lower hemitympanum, related to mid-frequencies (yellow arrow) via the inferior tympano-mallear connection (lower black dot).

The TM is a vibrating membrane. Ideal circular vibrating membranes possess many resonance frequencies, corresponding to the preferred modes. The fundamental (0,1) mode does generally not produce a clear and pleasing tone. Secondary modes (1,1), (2,1), (3,1), (4,1), (5,1), and sometimes (6,1) produce the most prominent overtones and are called the “preferred modes” (Figure 3A). The TM however is not an *ideal* circular membrane. It consists of an asymmetrical stiff “pars tensa” and a smaller flaccid “pars flaccida” (Figure 4); and it is attached to a closed tympanic cavity. Due to this specific anatomy, the resonance frequencies of the TM (TMRF) specifically connect with distinct locations on its surface (7–9). Maximum vibration patterns for the lower frequencies, roughly up to 1,000 Hz, are found on the upper quadrants; those for the mid-frequencies, roughly between 1,000 and 4,000 Hz, on the lower half of the TM. For the higher frequencies above 4,000 Hz the vibration pattern is complex.

Vibrations of the TM are transmitted to the malleus via two fibrous tympano-mallear connections (10). The TM thus consists of three distinct *functional units:* the *superior hemitympanum* preferentially transmits lower frequency sound waves via a superior tympano-mallear connection; the *inferior hemitympanum* preferentially transmits mid-frequency sound waves via an inferior connection. The *pars flaccida* forms a third functional unit.

TT, phylogenetically a masticatory muscle, innervated via the mandibular branch of the trigeminal nerve, inserts on the neck of the malleus handle, approximately at the level of the superior tympano-mallear connection: TT contraction therefore mainly influences the stiffness of the upper hemitympanum, related to the lower frequencies. TT function is largely unknown. It contracts after stimulation of certain facial areas (11, 12), on contraction of certain muscles (13, 14), as part of the startle reaction (15, 16), and on speaking or the intention to speak (16), during belching, yawning, and swallowing (17), but without contributing to ET opening (18).

Another area prone to modification by muscular action is the pharyngeal part of the ET and adjacent PR. Muscles innervated by the mandibular branch of the trigeminal nerve and the vagal nerve are involved: the tensor veli palatini (V) and levator veli palatini (X) muscles, the medial pterygoid muscle (V), the salpingopharyngeal muscle (X). Medially, the prevertebral or deep flexor muscles (cervical plexus) influence the shape of the PR. This muscular apparatus is believed to adjust middle ear pressure by opening and closing ET; but, for this assumed pressure related function (19), its lay-out is overly complex and lacks logic.

**Figure 5:**
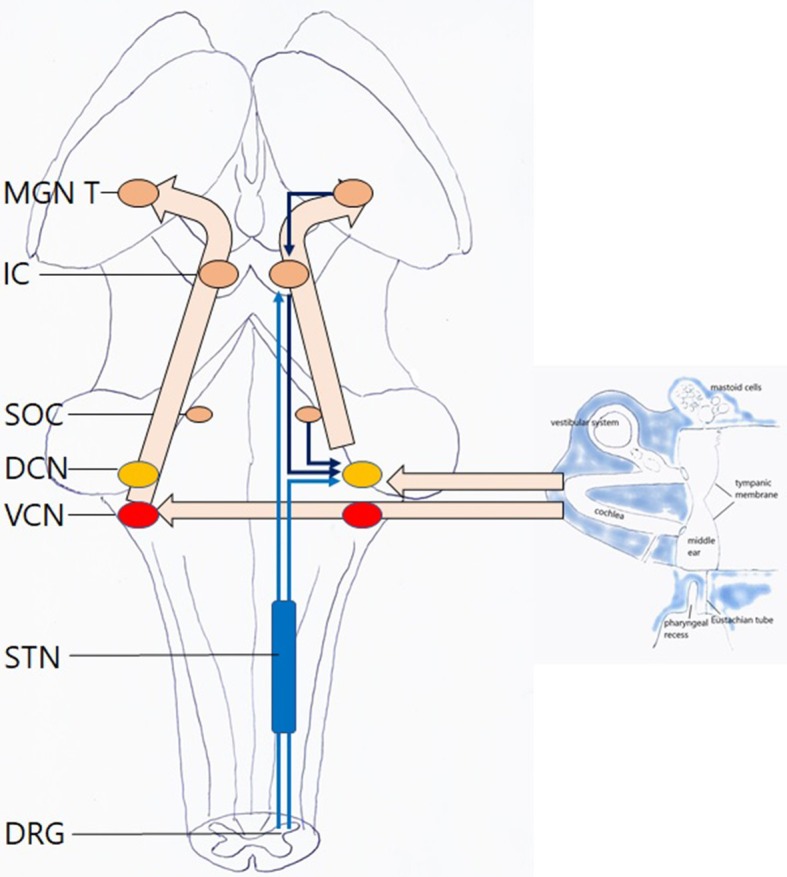
Hearing: this is an automatic, passive process. Left hollow arrows: ventral pathway via the VCN. Right hollow arrows: dorsal pathway via the DCN with afferent and efferent inputs. Light blue arrows: somatosensory input to the dorsal pathway, with cervical and trigeminal origin, via the trigeminal nuclei. Dark blue arrows: central input to the dorsal pathway MGN medial geniculate nucleus of thalamus; IC inferior colliculus; SOC superior olivary complex; DCN dorsal cochlear nucleus; VCN ventral cochlear nucleus; STN spinal trigeminal nucleus; DRG dorsal root ganglia. For clarity sake, midline crossings and several descending pathways have been omitted.

Once transferred toward the cochlea, sound waves are translated into electrical impulses that move through the cochlear nerve to the brain (Figure 5). The “classical” or “lemniscal” pathway via the ventral cochlear nucleus (VCN) conveys information on the *content* of the acoustic stimulus. The “non-classical,” “extralemniscal” pathway via the dorsal cochlear nucleus (DCN) conveys information on *attributes* of the sound that may be of value in assessing its safety, threat or emotional content. Attention modulation relative to bottom-up stimuli occurs via this pathway (20). Both VCN and DCN project to the superior olivary complex and inferior colliculus (IC) where integration from information from both ears allows for directional hearing; then to the thalamus where auditory content is integrated with content from other senses; and finally to the auditory cortex. The DCN also receives efferent innervation from the auditory cortex, superior olivary complex and IC; DCN and IC also receive proprioceptive and cutaneous, but not nociceptive, input from trigeminal and dorsal cervical root origin. The function of these connections was hypothesized “to suppress responses to ‘expected' body-generated sounds such as vocalization or respiration. This would serve to enhance responses to ‘unexpected' externally-generated sounds, such as the vocalizations of other animals” (21, 22).

## Hypothesis

In the music instrument called “kettle drum” or “timpani,” air compression in the bowl results in damping of the unwanted and disturbing fundamental (0,1) mode. The secondary modes can be influenced by adjusting the volume of the bowl and the stiffness of the drumhead. Correct tempering of this music instrument results in quasi-alignment of the preferred modes, so that a quasi-harmonic series of overtones is formed (Figure 3B). This series defines pitch, harmonicity, timbre, and clarity of the sound. Its pitch relates, not to the original damped fundamental corresponding to mode 0,1; but to a virtual “missing fundamental” (MF) located at ½^*^f0, f0 corresponding to the first preferred mode 1,1 (f0 or mode 1,1 = 2^*^MF; 2,1 = 3^*^MF; 4,1 = 4^*^MF; 6,1 = 5^*^MF, and so on).

Similarly, air compression in the closed tympanic cavity damps the (0,1) mode in the TM, and the body disposes of ways to adjust the volume of the cavity and the stiffness of the TM. A correct “tempering” results in alignment of the preferred modes to a quasi-harmonic series. This controllable system allows for the transfer of a clear, rich and full (quasi-harmonic) sound with highly intelligible content, when one wants to zoom in to a harmonic sound; or a dull, non-invasive sound when zooming out.

### Two Antagonistic Muscular Systems

**Figure 6:**
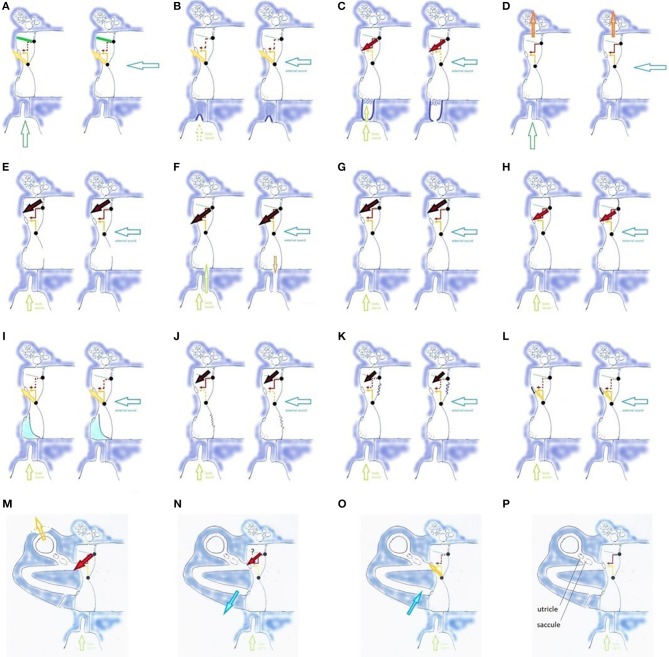
Influences on TMRF (physiologic mechanisms **(A–D,G,H)**, pathologic mechanisms related to middle ear **(E,F,I–L)**, and inner ear **(M–P)**; **(A)** TT contraction (TT: green line) shifts TMRF upward, decreased transmission of lower frequencies (dotted red arrow) and increased transmission of mid-frequencies (yellow arrow). Similar effect for pharyngeal (left, vertical hollow arrow) and external sounds (right, horizontal hollow arrow); **(B)** PR/ET closure shifts TMRF upward for pharyngeal and external sounds and decreases awareness of pharyngeal sounds (dotted vertical hollow arrow); **(C)** opening the PR/ET complex shifts TMRF downward for pharyngeal and external sounds (dotted yellow arrow, red arrow) and increases awareness of pharyngeal sounds; **(D)** filtering effect of antrum, mastoid and temporal apex cells. Specific frequency bands may be involved, depending on the vascular filling of these cells and gas composition in the mastoid. The effect may be different for pharyngeal and external sounds; **(E)** loss of the middle ear air cushion (here after paracentesis) causes a sudden and uncontrolled increase of the (0,1) mode and decrease of the preferred modes. A reactive TT contraction and PR/ET closure may shift TMRF upward (not illustrated). The large black arrow indicates the increase in very low frequency transmission, corresponding with the missing fundamental and the (0,1) mode; **(F)** loss of the air cushion in PET (extra vertical hollow arrow): increased pharyngeal sound transmission causes autophony; middle ear air cushion loss and its effect on the (0,1) mode causes dull hearing; **(G)** Pars flaccida loose: decrease of air cushion effect with downward TMRF shift; **(H)** Pars flaccida tense: increased air cushion effect, TMRF shifts upward from the very low to the low frequencies. The stiff pars flaccida acts like an extension of the upper hemitympanum, which causes an increased transmission for low frequency sounds (red arrow). **(I)** Partial filling of the tympanic cavity, causing TMRF upward shift. Compensatory PR/ET opening (not illustrated) may increase pharyngeal sound transmission and cause autophony; in more complex situations the fluid may impact against the TM or cover the RW; **(J)** Flaccid area in lower hemitympanum causes a partial elimination of the middle ear air cushion and greatly diminishes the transmission by the lower hemitympanum: both effects shift TMRF downward. In this long standing, chronic situation, one may expect compensation by the slow mechanisms, but resonance homeostasis may remain brittle, and certain events will cause symptoms; **(K)** Flaccid area in the upper hemitympanum. Partial elimination of the middle ear cushion effect, and decrease of transmission of the upper hemitympanum, which shifts TMRF upward. Compensation by the slow acting mechanisms may be expected, and decompensation by seemingly minor causes; **(L)** Otosclerosis: TMRF upward shift compensated by slow compensation mechanisms (not illustrated), resonance homeostasis remains strong; **(M)** Third window lesions (dehiscence or near-dehiscence); **(N,O)** Acute intracranial hypotension/hypertension; **(P)** Saccule and utricle.

**Figure 7:**
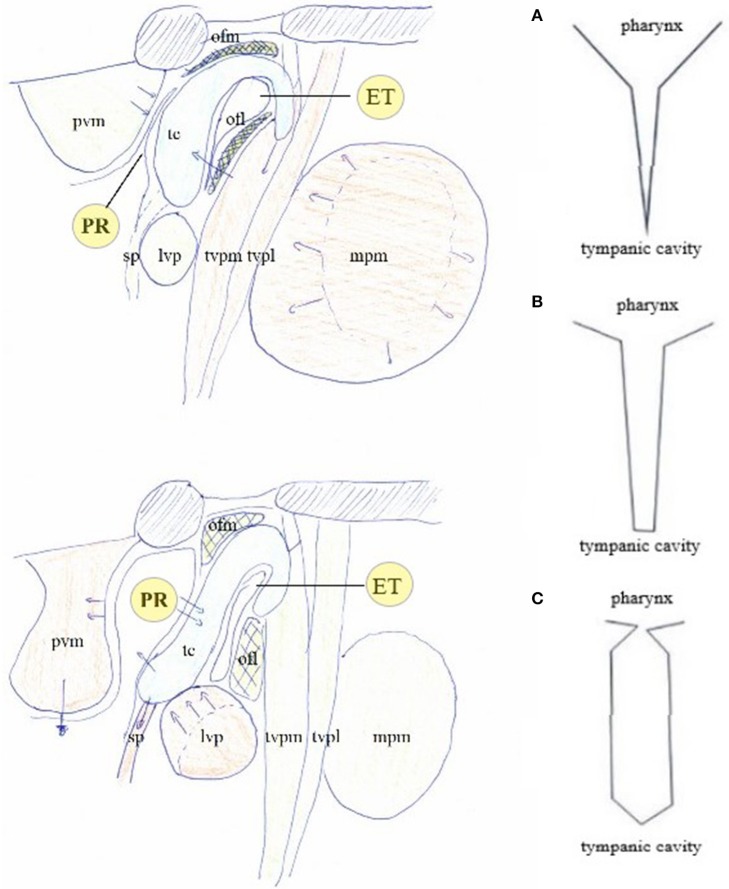
The muscular apparatus modifies the shape of the PR/ET complex. Contraction of trigeminally innervated muscles with relaxation of vagally/cervical plexus innervated muscles closes PR/ET complex (upper left); contraction of vagally/cervical plexus innervated muscles with relaxation of trigeminally innervated muscles opens PR/ET complex (lower left) **(A)** PR/ET closed; **(B)** theoretical neutral position; **(C)** PR/ET opened. Pvm, prevertebral muscles (cervical plexus); PR, pharyngeal recess; tc, tubal cartilage; sp, salpingopharyngeus muscle (X); lvp, levator veli palatini muscle (X); ofl, lateral Ostmann's fat pad; ofm, medial Ostmann's fat pad; tvpm/tvpl, medial and lateral layer of tensor veli palatini muscle (V); ET, Eustachian tube lumen; mpm, medial pterygoid muscle (V).

Two muscular systems, innervated by the mandibular branch of the trigeminal nerve and the vagal nerve, exert an antagonistic effect on TMRF. The direct influence acts via the TT muscle: by its stiffening effect of the upper half of the TM, TT contraction shifts TMRF for the lower frequencies upward (23–26) (Figure 6A). The indirect influence on TMRF is hypothesized to act via the PR/ET complex (27) (Figure 7). Contraction of the muscles innervated by the vagal nerve and anterior cervical plexus, coordinated with relaxation of the trigeminally innervated muscles, elongates and widens the PR fundus while closing its entrance, and stretches the fat in OFL and OFM. Acoustically, this space is hypothesized to become an extension of the cavity. This “volume increase” of the cavity is thought to decrease TM stiffness, shifting TMRF downward. Experience with patching suggests that this probably mostly relates to the mid-frequencies. Also, sounds originating in the pharynx are now allowed to travel freely to the middle ear, to be captured by the TM and transmitted to the cochlea. Relaxation of the vagally/cervical plexus innervated muscles and contraction of the trigeminally innervated muscles, opens up PR entrance, closing its fundus while firmly compressing the fat pads. The elimination of the virtual extension of the middle ear cavity is now hypothesized to increase TM stiffness and shift TMRF upward. Also, awareness of pharyngeal sounds diminishes.

In this hypothesis, TT contraction/relaxation and PR/ET complex closure/opening (Figures 6B,C) shift TMRF in a synergistic manner. This dual trigeminal/vagal mechanism allows for both a gradual and controlled TMRF modulation and body sound awareness.

### Other Influences on TMRF

#### Helmholtz Resonance

**Figure 8:**
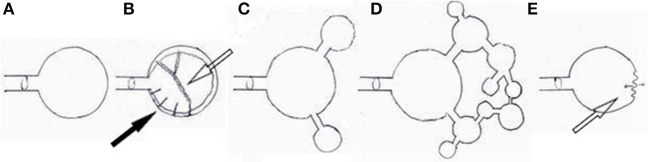
Theoretical models. **(A)** simple Helmholtz resonator; **(B)** “resonator with variable absorbing lining material:” damping by mucous membranes, mucosal folds hanging across the tympanic cavity (hollow arrow), and baffle-like open hypotympanic cells on the tympanic cavity floor (black arrow); **(C)** “combination of multiple resonators:” simplified concept in which the attic, mastoid, and temporal apex, act as extra resonators that absorb specific frequencies from the main resonator; **(D)** “resonator tree:” more realistic concept, in which a multitude of small air cells (small resonators) ultimately alters the damping properties of the whole system in a complex way; **(E)** “Compliant Backplate Helmholtz Resonator:” resonance of the system is modified by the stiffness properties of the TM (hollow arrow), that transmits part of the energy outside the resonator.

Reflecting waves in the cavity influence TM vibrations and thus TMRF (28). In a *simple Helmholtz Resonator* (Figure 8), RF is defined by the formula $f_{H}=\frac{v}{2\pi }\sqrt{\frac{A}{V_{0}L}}$. *A* and *L* stand for diameter and length of the neck of the resonator, respectively, and *V0* for the volume of the body of the resonator. *RF thus shifts upward with a larger neck diameter, shorter neck, and smaller cavity*. TM is the compliant backplate, PR/ET the neck, the middle ear cavity the lumen, lined with the mucous membranes and hypotympanic cells; and linked to the attic and the complex maze of distal mastoid and apex air cells, of which the effect on TMRF can be quite diverse.

Examples of Helmholtz Resonance issues are: a decrease in cavity volume when the cavity is partly filled with fluid shifts TMRF upward (“simple Helmholtz resonator”). Localized congestion in the passageway between the middle ear and mastoid (the aditus), eliminates the damping effect for specific frequencies (“combination of multiple resonators”). In individuals with vulnerable resonance homeostasis, mucosal congestion of an isolated cell in the distal mastoid or temporal bone apex may lead to unexpected complaints (“resonator tree”) (Figure 6D) The body can exert control on TMRF via Helmholtz resonance related mechanisms: vascular filling of parts of the mastoid (29) and shape of ET (in otosclerosis: see further in the text).

#### The Middle Ear Air Cushion Effect

Trapping of air inside the cavity increases TM stiffness and shifts TMRF upward; this is most prominently expressed in the damping of the (0,1) mode and expression of the preferred modes (Figure 3). When a tiny opening is created in the cavity wall, this middle ear air cushion is eliminated, the (0,1) mode revives and the preferred modes are damped. This results in a brutal and uncontrolled downward shift of TMRF (30). This all-or nothing phenomenon occurs immediately after traumatic perforations of the TM, after paracentesis (Figure 6E), in Patulous Eustachian Tube syndrome (PET, a condition in which the ET is continuously wide open) (Figure 6F).

The body may make use of the air cushion effect in order to modulate TMRF in a controlled way: modulating middle ear air pressure relative to atmospheric pressure (29) increases the stiffness of the ordinary loose (Figure 6G) pars flaccida and gradually enhances the air cushion, causing a gradual upward shift of TMRF (31, 32) (Figure 6H).

#### Standing Waves on the TM (33, 34)

It is not clear what causes them, and whether they have a function, or should be considered as a nuisance. A system capable of altering TM stiffness might be able to produce, eliminate, and use them.

### Feedback Loop and the Concept of Resonance Homeostasis

A “Tympanic Resonance Regulating System” (TRRS) is hypothesized to consist of a *sensor* that measures the present situation, a *controller* that decides on the need for RF shift, and prompts the *actuator* to change resonator properties.

There are slow, medium, and fast **actuators. Two slow acting mechanisms**, varying over years to decades, are based on growth of anatomical structures: mastoid pneumatization and ET cartilage anatomy and consistency. They provide an optimal underlying basis for the “resonance homeostasis,” on which the medium and fast acting mechanisms superimpose their modulating effects. If this basis is sound, minimal straining of the resonance regulating system is needed to guarantee homeostasis conservation and use of the zooming function. The slow mechanisms adapt the system to specific anatomical properties of the head and upper airways, the acoustic properties of one's voice, the effects of inherited disease and perhaps long-standing external circumstances. Two **moderately slow acting mechanisms**, acting within hours, are based on vascular mechanisms. Congestion of mucous membranes in the tympanic cavity increases damping (“variable lining material”), and in specific areas of the mastoid and apex, allows for filtering and elimination of specific frequencies (“resonator tree”). Inducing a slightly negative or positive middle ear pressure (32) increases pars flaccida stiffness (air cushion). They adapt TMRF to specific atmospheric conditions, body position, the diurnal rhythm. Two **fast actuators**, TT and PR/ET, provide a quick response mechanism based on a muscular mechanism. They act within fractions of a second and maintain their action for minutes, hours, days as necessary, responding to changing acoustic circumstances, to allow for the zooming function, and to modify pharyngeal sound awareness.

Peripheral **sensors** monitor TMRF. Tympanic plexus baro- and chemoreceptors (35, 36) trigger the moderately slow actuators. TM stress receptors (37, 38) and Ruffini corpuscles in the PR fundus and posterior nasopharyngeal wall (39) trigger the fast actuators. Theoretically, acoustic clues gathered via the cochlea, proprioceptive receptors in the muscles, and sensors on other locations may be involved.

The **controller** function for the *upward shifting system* is proposed to be located in the trigeminal nuclei and dorsal root ganglia: the afferent path running from sensors on the TM and the masticatory muscles via the auriculotemporal nerve, branch of the mandibular branch of the trigeminal nerve. The efferent path toward the actuators consists of the mandibular branch of the trigeminal nerve. As for the controller function for the *downward shifting system*: receptors on the TM may send information via the auricular branch of the vagal nerve and the great auricular nerve to the solitary nucleus, the principal sensory trigeminal nucleus, spinal trigeminal nucleus and cuneate nucleus (40, 41). The efferent pathway then consists of the pharyngeal branch of the vagal nerve and the ventral rami of the cervical plexus (and probably the IX). The controller is under the influence of central inputs, related to anxiety and mental stress [hypothetical cochlear sensors would send their information via VCN or DCN toward the spinal trigeminal nucleus (42)].

### Trigeminocervical Complex

**Figure 9:**
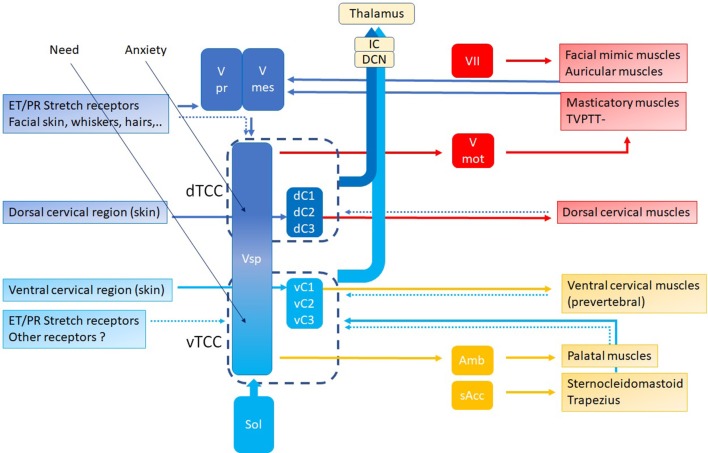
TCC with dTCC and vTCC. Vpr Principal or Chief Trigeminal nucleus. Vmes Mesencephalic Trigeminal nucleus. Vsp Spinal Trigeminal nucleus. Vmot Motor Trigeminal nucleus. Sol Solitary nucleus Amb nucleus Ambiguous. sAcc Spinal Accessory nucleus. Dark blue arrows: afferent, dorsal system, Light blue arrows: afferent, ventral system (proprioceptive, exteroceptive); red arrows: efferent dorsal system, yellow arrows: efferent ventral system (motor). Dotted arrows indicate unproven pathways. Vpr and Vmes have not been duplicated in vTCC.

In this hypothesis, the controller is the “Trigeminocervical Complex” (TCC) (43, 44): a well-known functional entity located at the trigeminal nuclei level, including sensory and motor elements from cervical nerves and trigeminal nerve, that explains the co-occurrence of pain and muscle tensions in regions innervated by these nerves. TCC output is peripheral, related to muscular and other functions that shift TMRF, and central, related to modulating the incoming (acoustical) information. If TCC is to be the controller of the resonance regulating system, it necessarily consists of two components, related to upward and downward shifting (Figure 9), respectively, the tTCC or dTCC (“trigeminal” or “dorsal” TCC, related to trigeminal nerve and dorsal cervical nerves), and vTCC (“vagal” or “ventral” TCC, related to vagal nerve and cervical plexus).

**Figure 10:**
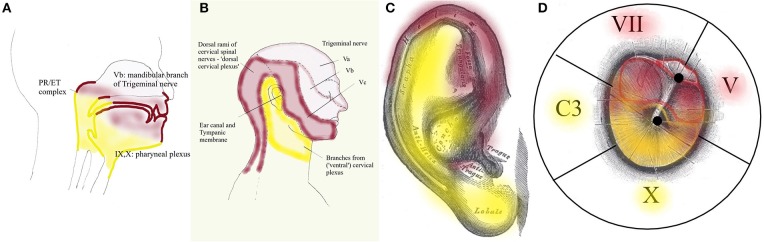
Dermatomes innervated by dTCC (red) and vTCC (yellow). The “demarcation line” runs in the pharynx **(A)** through the PR/ET complex, nasopharyngeal region, palate, tongue base; externally **(B)** along the mandibula, through the pinna (from Gray's anatomy via Wikipedia) **(C)**, external ear canal and TM (propriocepsis of the mimic muscles is carried by the Vth nerve) **(D)**. Symptoms related to dTCC/vTCC activation and muscular tensions rigorously respect these dermatomes.

Peripheral input for dTCC comes from sensory and motor structures, innervated by the dorsal rami of the cervical nerves. These carry sensation of the dorsal neck region innervated by the greater occipital nerve (C2) and motor function of the dorsal paravertebral neck muscles (splenius capitis, semispinalis capitis, …). The trigeminal origin includes sensation of the region innervated by the trigeminal nerve. In the context of this hypothesis, mostly the mandibular branch of the trigeminal nerve (ear canal, TM, latero-anterior part of the tongue, face), and to a lesser degree the other branches; and proprioception from the masticatory, tensor veli palatini and TT muscles. The mimic and auricular muscles take part in dTCC input: their proprioception is carried by the trigeminal nerve. Central input mainly relates to anxiety. Inputs in any part of the system may trigger dTCC activation: dental problems causing masticatory muscle tensions, anxiety, the need for a TMRF upward shift. dTCC output activates trigeminally innervated muscles, and other peripheral upward shifting mechanisms; and other trigeminally innervated organs aimed at detecting unpredictable events. dTCC activation may cause complaints along the dermatomes innervated by C2 and the trigeminal nerve (mainly its mandibular branch) (Figure 10): otic symptoms (45), dorsal cervical and masticatory muscular tensions, tension type headaches, trigeminal pain syndromes, burning mouth syndrome, headaches related to dorsal C2.

Following the logic in this hypothesis, vTCC input then is expected to contain afferent inputs from the vagal, glossopharyngeal and accessory nerves, and cervical plexus. Sensory input consists of sensation of the tympanic membrane, pinna, and ear canal via the auricular branch of the vagal nerve, part of the (naso-)pharynx via the pharyngeal plexus, the trigger-point of the superior laryngeal nerve, and the region between ear and hyoid; proprioception from the trapezius, sternocleidomastoid (SCM), and prevertebral muscles; and central inputs related to moods (mainly need). Efferent outputs may be carried via the vagal nerve and cervical plexus toward prevertebral muscles, suprahyoid and infrahyoid muscles, trapezius, SCM, levator scapulae, scalenus medius (cervical plexus); and the muscles of palate and pharynx except stylopharyngeus and tensor veli palatini (pharyngeal branch of the vagal nerve). The hypothesis assumes that inputs in any part of the system may trigger vTCC activation: trapezius muscle tensions due to posture problems, shoulder problems after a fracture, sensitization of the vagal system after chemotherapy, mental stress related to the family situation, the need for a downward TMRF shift. vTCC output causes contraction of vagally innervated muscles and other mechanisms related to downward shifting, and organs aimed at optimizing the sensation of predictable stimuli. vTCC activation may cause sensory symptoms along the dermatome innervated by the auricular branch of the vagal nerve and the pharyngeal plexus (Figure 10): i.e., “otic” symtoms, and/or the symptoms of “sensory laryngeal neuropathy” (lump feeling, throat pain, swallowing problems, feeling of slime in throat, …), accompanied by pain in the muscles that are proprioceptively innervated by the cervical plexus (most prominently in trapezius and SCM muscles).

Patching the upper hemitympanum increases its stiffness, damps lower frequencies, eliminates the need for contraction of TT; it decreases dTCC activity, possibly by deactivation of stretch receptors on the upper quadrants. Patching the lower hemitympanum damps the mid-frequencies, eliminates the need for contraction of vTCC related muscles; it decreases vTCC activity, probably by deactivating stretch receptors on the lower hemitympanum and PR/ET. It is a “tympanic desensitization.”

### Physiology

#### Zooming Function of the TRRS

Peripherally, TRRS may favor perception of certain sounds by shifting TMRF in the frequency domain. Arguments for this putative mechanism can be found in the experiments involving TT contraction (23–26). It may function by increasing or decreasing quasi-harmonicity of the preferred modes. “Straining our ears” allows us to perceive desired harmonic sounds as clear, and undesired harmonic sounds as dull and lacking pitch. Arguments are similarities with the kettle drum or timpani. The zooming function may also function in the time domain: sounds may be perceived clearly when the transfer function is stable over time, and as blurred when it is highly variable. Arguments are similar findings concerning hearing and vision. Other peripheral mechanisms may be possible.

Attempts to measure these effects have until now failed to provide evidence, and, similar to what is seen in other muscular systems (46), the peripheral function may be only vestigial. The slight low frequency hearing loss however that is seen very often in this patient group, suggests a real effect on TM stiffness.

**Figure 11:**
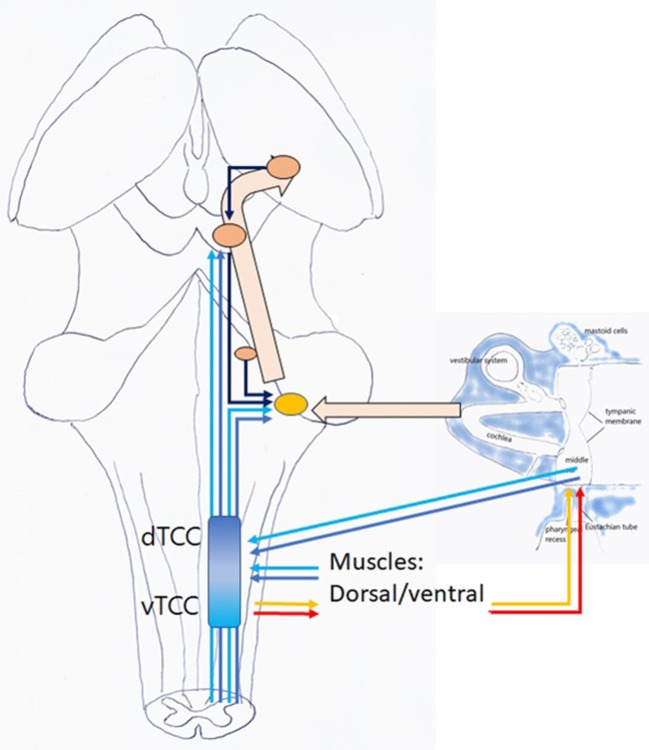
Listening: this is an active, voluntary process (unconscious or conscious). TCC with afferent dTCC (dark blue) and vTCC (light blue); efferent TMRF upward (red); and downward shifting (yellow).

Centrally, zooming may be achieved as TCC activation increases activity at the DCN and IC level, increasing hearing acuity and directional hearing (21) (Figure 11). Moreover, modification of the throughput may alter the perception of acoustic input, and influences responses at the level of the higher neural networks. Other central mechanisms are possible.

#### Directing and Modulating of Auditory Attention (=Listening)

When a top down or bottom up stimulus incites the mammal to listen to acoustic information related to unpredictable events, dTCC is activated. Peripherally, mastication stops, dorsal cervical muscle contraction extends the face with its antennae toward unpredictable stimuli (21); PR/ET closure may decrease pharyngeal sound awareness; and TMRF may shift upward while its variability decreases. Centrally, increased DCN and IC activity sharpens *ipsilateral* hearing acuity, and directionality (21). Probably, an effect occurs via the efferent bundle toward the outer hair cells in the cochlea. Further away, simultaneous contraction of spinal and extra-ocular muscles produces a perfect standstill; and antennae (whiskers, skin, vision, smell, …) are activated. The function of other organs may be inhibited (rummaging from stomach). Central networks may be activated.

When incited to monitoring predictable sounds related to domestic tasks and communication, it is proposed that vTCC be activated. Peripherally, vagally and cervical plexus innervated muscles contract. The face bends downward (toward source of food, children, and predictable tasks), PR/ET opens, TMRF shifts downward while its variability decreases. Centrally, DCN and IC activation enhances *bilateral* awareness of sounds, and the efferent bundle is activated. Further away, limb-girdle muscles contract, other organs such as the vestibular and baroreceptors, and senses at the level of mucous membranes, taste, internal organs, are activated, while exteroception at the trigeminal level is inhibited. Central networks may be activated.

#### Sensation to Perception

Acoustic input reaching TCC is still “sensation:” raw, unprocessed, high-fidelity data. In TCC, this input is modified, relative to the somatosensory and central input, which in this hypothesis depends on the dTCC/vTCC predominance. This transition through TCC thus forms the first step in the transformation from “sensation,” being a high-fidelity recording of the external world, to “perception,” a functional experiencing of the external world, colored by one's own experiences, predictions, emotions. This altered perception enables the higher neural networks to respond more accurately, e.g., to unpredictable vs. predictable events. In this hypothesis, acoustic stimuli traveling through a dTCC dominated TCC may be perceived differently than vTCC-modulated stimuli. Interestingly, this assumption means that transformation from sensation to perception at the TCC level is not solely influenced by central but also by somatosensory input: cutaneous stimuli (47) and muscular tensions color the perception of the world.

dTCC is activated in situations of real or imagined danger, when *alertness, and scanning* ones surroundings is important: it is the “*outdoors component*” of TCC; vTCC may rather be activated when one has to tend to physical need (looking for food), emotional need and/or the needs of the family (communication); when *concentration and focus* are important: it is the “*domestic component*” of TCC.

#### Further Effects

TCC forms a pivotal integration center between “body, mind, and the external world.” Its three inputs (central, somatosensory, and external), influence its outputs (peripheral in muscle tonus and activity of the sensing organs, central in attention, and perception). This cannot else than have a profound influence on emotional states, sympathetic/parasympathetic balance, hormonal elements such as the hypothalamic-pituitary-adrenal axis, cardiovascular regulation, … An example is the similarity between vTCC and the “social engagement system” of the Smart Vagus (48). In contrast to vTCC, which is defined in the framework of an attention and perception related system, the social engagement system is largely described from an afferent point of view. But they are probably two very closely related and overlapping systems.

In a somewhat exaggerated and simplified portrayal, one could characterize patients with dTCC activation as filling the consultation room with vibrant energy; standing upright, their eyes explore the surroundings, ready for action. On palpation hard dorsal neck and masticatory muscles. High anxiety. The typical vTCC patient would be pictured as accompanied by children or spouse, sitting with a forward head posture, shoulders down. Prevertebral, SCM and trapezius muscles painful on palpation. This caring person has (emotional) needs, carries the world on his shoulders. His battery is low.

An implication of these statements is that long-standing dTCC activation may in the long term modify the functioning of the brain and cause body dysfunction; that disturbances in tympanic resonance may induce anxiety by bringing a pre-existing subclinical anxiety over a certain threshold. A rather far-reaching prediction is that people with a brittle, vulnerable resonance homeostasis, because of e.g., a third window or flaccid area in the TM, would be more prone to develop anxiety.

### Pathology: “Tympanic Dissonance Syndrome”

#### Pathophysiology

In this hypothesis, “Tympanic dissonance” indicates a pathological condition, where any form of TRRS malfunctioning produces many combinations of symptoms, via two underlying mechanisms (Table 1). The first putative mechanism consists of an increased TCC activation, bottom-up by a strained TRRS or top-down by central inputs. The zooming function may be impeded, and/or it may be preserved at the cost of TCC activation related symptoms. The second mechanism proposed, consists of decreased TCC thresholds, leading to an exaggerated peripheral (local or remote) or central response in a perfectly normally functioning TRRS. Often symptoms arise only when several mechanisms are present, and disappear as one of the mechanisms is tackled. The hypothesis e.g., implies that a third window or PET need only be operated if tympanic patching combined with psychological treatment does not eliminate the symptoms. Often, underlying resonator disturbances [e.g., a third window, a long-term increased listening effort (49)] exert a longstanding pressure on the baseline resonance, compensated at the cost of seemingly unrelated symptoms. Decompensation then follows a minor extra shift (e.g., inflammation, a long car drive, mental stress, and anxiety), which would easily be handled in a perfectly balanced system. Symptomatic or etiological treatment may restore resonance homeostasis and eliminate the symptoms. Over time however, central feedback mechanisms based on anxiety, or sensitization can occur, and these can better be addressed *prior* to treatment of the initial dissonance cause.

**Table 1:** Pathophysiologic mechanisms.

Inappropriate controller (TCC) activation.
1. Increased TCC input. 1.1. Peripheral from sensor: overcharging of zooming function 1.2. Peripheral from resonator: problems with resonator, slow/medium actuators 1.3. Peripheral from actuator: problems with fast actuators 1.4. Central: neurological/mental
2. Decreased TCC tresholds


##### TCC activation through increased input (strained TRRS)

*Increased peripheral TCC input related to the sensor.* Straining by a sustained listening effort occurs when acoustic circumstances are difficult (prolonged phone use in call centers, radio listening during long car drives), in hearing loss (e.g., noise trauma), and in hypervigilance (anxiety in imagined or real danger; e.g., in posttraumatic stress disorder). These patients are constantly scanning their surroundings for possible threats.

*Increased peripheral TCC input related to resonator and slow/medium actuator problems.* Such underlying problems cause a brittle resonance homeostasis, which is easily disturbed, and calls for increased and repeated reaction and straining of the fast actuators.

Resonator neck problems can be PR/ET adhesions, cysts, tumors, inflammation. Hindering the closing mechanism might cause ipsilateral autophony with symptoms of ipsilateral dTCC activation and trigger points in the ipsilateral masticatory/dorsal neck muscles. Hindering the opening mechanism might cause bilateral or contralateral autophony with bilateral symptoms of vTCC activation and bilateral pain on palpation of the prevertebral, SCM, and trapezius muscles (27).

Resonator body problems may occur when the middle ear is partially filled (50) (Figure 6I), or local inflammation eliminates the secondary mastoid resonators.

Resonator wall problems can be diverse. Thinner, “flaccid” areas on the TM (Figures 6J,K) have a location-independent downward shifting effect (air cushion decrease), and a location-dependent damping effect on TMRF. Therefore, in Tympanic Dissonance, patching a flaccid area wherever it is located, is bound to almost always produce the desired effect (51). Localized inflammation in the lateral posterior attic, at the level of the lateral incudal fold between the short process of the incus and the lateral attic wall, is a specific clinical entity. In patients in whom TCC was already firmly activated through muscular tensions or anxiety, the upward shift of the TOS RF and alteration of the extra resonator may provoke symptoms and lead to vicious circles of anxiety and stress. In fenestral otosclerosis, increased stiffness of the annular ligament of the stapes footplate is expected to increase TOS stiffness, and shift TMRF upward (Figure 6L). Tympanic Dissonance symptoms do not develop in this often hereditary condition, as the slow and moderately slow mechanisms provide a compensatory downward shift. During surgery however, TOS stiffness decreases dramatically: the symptoms that typically last during some 3 weeks after this abrupt re-calibration much resemble Tympanic Dissonance symptoms. An interesting question is whether the compensating RF downward shift could influence the typical butterfly-like audiometric curve and the Carhart notch. Also related to the resonator wall are modifications of the cochlear load. In third window syndromes (e.g., superior semi-circular canal dehiscence or SSCD), autophony, other body sounds, some types of vertigo, fullness feeling in the ear, and tinnitus (52, 53), may be related to a recent failure of compensation (Figure 6M). Acute intracranial hypotension provokes autophony (54) (Figure 6N), and intracranial hypertension pulsatile tinnitus (55) (Figure 6O); both can cause vestibular and sensory symptoms as well. Symptoms fade when the condition becomes chronic, probably as the moderately slow acting compensating mechanisms establish a new resonance homeostasis.

Middle ear air cushion related complaints occur in PET (cfr infra). In “resonance prone patients,” paracentesis may produce annoying complaints, that diminish when a grommet is placed in the opening (increase in TM mass and stiffness); even more when a paper patch is placed over the lumen of the grommet (restauration of air cushion). In these patients, accumulation of keratin around the grommet may result in disturbing complaints, mainly pulsatile tinnitus [even small hair cells on the TM may cause disturbing symptoms (56)!].

*Increased peripheral TCC input relating to the fast actuators.* Bruxism due to bite problems or dental pathology, and dorsal neck tensions secondary to cervical spine pathology shift TMRF upward and cause dTCC activation. Posture problems, cervical spine pathology, Trapezius tensions, shoulder problems, are proposed to shift TMRF downward and cause vTCC activation.

*Increased central input.* Increased central input causes inappropriate TCC activation hence output, and subsequent TRRS dysfunction. Neurologic brainstem hyperexcitatory conditions are rare (perhaps neurovascular conflicts of the cochlear and/or trigeminal nerve could fit in this category). Mental factors however are ubiquitous in these patients, and particularly anxiety and mental stress/emotional neediness need to be addressed in dTCC/vTCC activation, respectively.

##### TCC activation through decreased thresholds

In peripheral or central sensitization, local, or remote symptoms can be caused by exaggerated responses to normal inputs in a perfectly normal functioning TRRS. In these patients, a flaccid area on the TM may trigger burning mouth, PR inflammation pulsating tinnitus or disequilibrium, a shoulder problem hyperacusis—it's up to the clinician to find which normal input triggers the exaggerated output. It seems that the more sensitization, the less resonator disturbances are necessary to provoke the more widespread complaints. When sensitization is strong, tympanic patching, in our clinical experience, sometimes produces clear effects on distant complaints.

The resemblance of the “sore throat/painful lymph node” phenotype of Chronic Fatigue Syndrome (57) with the description of vTCC activation is remarkable: sore throat (actually laryngeal sensory neuropathy), so-called “swollen lymph nodes” (actually no lymph nodes, but sensitivity of the superior laryngeal nerve) and dizziness (saccular excitation type). In as yet unpublished clinical experience, patching appears to provide good and stable results for throat complaints and dizziness; the results often last some weeks to months after the patch has disappeared. In contrast, in fibromyalgia patients the activation appears to be variable: ordinary day-to-day resonance regulation causes severe complaints, effectively eliminated by tympanic patching. A few days later some event (change of the weather, wrong movement of the neck, …) causes a need for a TMRF shift in another direction; symptoms suddenly and acutely return, as the patch now increases TCC activity instead of decreasing it! Patch removal calms down the symptoms, and the beneficial effect is obtained again by patching another location, until some minor event again reverses the TMRF shift needed for resonance homeostasis (unpublished experience on relatively few cases).

Are some cases of Menière's disease related to Tympanic Resonance? Exactly the same symptoms appear, in acute and severe attacks. There is often a mental factor; atmospheric conditions may play a role, and grommet insertion (TM stiffness and weight, air cushion) provides a treatment option in some cases.

#### Symptomatology

Symptoms appear in varying clusters of auditory, vestibular, sensory, muscular, and perhaps central symptoms, grouped in a dorsal (dTCC) and a ventral (vTCC) cluster (Table 2).

**Table 2:** Symptom clusters.

**Tympanic dissonance: symptom clusters**
**1. Zooming dysfunction: auditory symptoms** 1.1. Body sounds 1.2. External harmonic sounds
**2. Normal zooming function: dTCC/vTCC symptoms** **2.1 Vestibular symptoms:** 2.1.1. dTCC: inappropriate utricular stimulation (less frequent) 2.1.2. vTCC: inappropriate saccular stimulation **2.2. Sensory symptoms (fine touch/vibration; proprioception)** 2.2.1. dTCC: trigeminal nerve and dorsal C2 dermatomes 2.2.2. vTCC: pharyngeal and cervical plexus **2.3. Muscular symptoms:** 2.3.1. dTCC: dorsal cervical, masticatory 2.3.2. vTCC: prevertebral, Trapezius, SCM, … **2.4. Other:** Tinnitus, central symptoms

##### Auditory symptoms

Auditory symptoms (Table 3): increased awareness of body sounds and/or external sounds, or on the contrary muffled hearing, reflect the deficiency of the zooming function. Decreased awareness of *body sounds* mostly goes unnoticed; increased awareness however can be very disturbing: autophony, pulsatile tinnitus, other sounds. Moreover, inappropriate TCC activation distorts not only the perception of the sounds, but also, at a higher level, emotional and other responses. Not only are these sounds overly loud, but they are disturbing and obnoxious, and trigger all kinds of undesired responses at several levels in the mind and body.

**Table 3:** Auditory symptoms.

Autophony	1. Other causes: Patulous Eustachian tube (PET) 2. Tympanic dissonance: 2.1. Increased TCC input 2.1.1. Resonator: neck: PR/ET dysfunction; cavity: partially filled cavity, extra resonator impairment; wall: third window syndromes (SSCD), intracranial hypotension. 2.1.2. Actuator: muscular tensions (e.g., stress) 2.1.3. Central: anxiety related feedback loops activate TCC, and produce hyperfocus on the symptom. 2.2. Decreased TCC tresholds: neuropathic conditions, sensitization, brainstem pathology.
Vascular sounds	1. Other causes: increased vascular sound level (cfr text) 2. Tympanic Dissonance: 2.1. Increased TCC input: 2.1.1. Resonator: cavity: otitis media with effusion; wall: intracranial hypertension, ear wax impaction. 2.1.2. Actuator: muscular tensions (e.g., stress) 2.1.3. Central: 2.1.3.1. Neurologic: brain stem problems, V and VIII vascular loops 2.1.3.2. Mental: hyperfocus, with persisting, anxiety related, feedback loops 2.2. Decreased TCC tresholds: neuropathic/sensitization: e.g., fibromyalgia.
Hyperacusis	1. Other causes: central, cochlear? 2. Tympanic dissonance: 2.1. Increased TCC input: inability to zoom out, to blur sounds 2.1.1. Resonator: without anxiety 2.1.2. Actuator: muscular tensions (e.g., stress) 2.1.3. Central: hyperfocus, with anxiety related feedback loops 2.2. Decreased TCC tresholds (neuropathic/sensitization): sudden and temporary symptoms following an ordinary trigger.
Muffled Hearing	1. Other causes: central, hidden hearing loss? 2. Tympanic dissonance: 2.1. Decreased TCC activation from faulty input: 2.1.1. Peripheral, resonator: e.g., elimination of air cushion. 2.1.2. Peripheral, actuator: muscle fatigue, due to increased listening effort 2.1.3. Central: depletion of neurotransmitters after noise exposure/prolonged listening? 2.2. (Increased TCC tresholds: does probably not exist)

**Autophony** *in PET* occurs mostly in women, and is related to hormonal changes, weight loss, and mucous membrane atrophy. It is not accompanied by sensory symptoms or muscular tensions; tympanic patching has no effect. Treatment consists of hormonal therapy, weight gain, ET narrowing surgery, or plugs. Autophony *in Tympanic Dissonance* is accompanied by sensory symptoms (most prominently fullness feeling) and muscle tensions. Anxiety may be present. Tympanic patching provides excellent results (1, 2); as do surgically thickening of the TM (58) or an etiological treatment (e.g., SSCD plugging).

Pulsatile tinnitus, or the **increased perception of vascular sounds on the heart rhythm**, is often attributed to an increased vascular sound level, caused by vascular or anatomic anomalies, e.g., high riding jugular bulb, aneurysma, carotid, or sigmoid sinus dehiscence, arteriovenous malformation, …. In the light of hypothesis however it may also be caused by a normally occurring vascular sound that cannot be eliminated by a malfunctioning TRRS. When diagnostic tests for increased vascular sound level have proven negative, one should focus diagnostics toward causes for Tympanic Dissonance and/or central amplification. Even if a cause for an increased vascular sound level is found, one should still bear in mind that most anatomical causes for such increased sound level have been present since birth, and may have been handled by the TRRS until the latter decompensated.

**Other body sounds** include cracking and clicking sounds on swallowing, hearing one's footsteps or the movement of the eyes, hearing rhythmic sounds. In spasms of the TT and tensor veli palatini one may look for signs of dTCC activation; in spasms of levator veli palatini for vTCC activation.

Increased awareness of *external* sounds (some forms of **hyperacusis**): in a strained TRRS, the ability to make sounds duller or to zoom out for sounds is impeded. The patient is obliged to listen to some sounds: at the restaurant, he is unable to understand his table partner and at the same time obliged to follow a conversation three tables further away. In sensitization, normal TRRS responses produce exaggerated effects. Moderately loud sounds or sustained listening effort provoke sudden and temporary peripheral effects such as pain in the ear, hearing a second sound, or a muscle spasm; or central effects of increased loudness or distortion due to an increased input in the DCN (21). Again, central elements and vicious circles of fear, anxiety, stress may be involved.

Some patients on the contrary complain of **muffled hearing**. Typically several audiometries have been done, showing normal hearing: hearing thresholds are normal indeed, but their hearing is dull: it is about quality, not quantity. Patients never report a “better,” but a “clearer” hearing after patching. Future research may tell if these could be related with some cases of “obscure auditory dysfunction,” “hidden hearing loss” or King-Kopetzky syndrome (59).

##### Symptoms related to the effects of TCC activation

*Vestibular symptoms.* Vestibular symptoms are often encountered in patients presenting with Tympanic Dissonance, and in our experience often respond well to patching (unpublished material). These relate to utricular and saccular function: linear acceleration in the horizontal or vertical plane is involved (walking, standing up, sitting down, …) and the complaints are non-rotatory (unsteadiness, falling when walking, veering to right or left, but also visus-related items such as oscillopsia, visual lag, …). The utricle and saccule are two small organs composed of thin membranes that hang loosely in the perilymph, the fluid contained in the labyrinth. Utricle is activated by movements in the horizontal plane, saccule by vertical plane movements. Both organs can be excited by sound presented to the ear or the skull. In VEMP testing, this excitation is evaluated indirectly by measuring its secondary effects: utricular excitation increases activity of the extra-ocular muscles, saccular excitation of the sternocleidomastoid muscle. The saccule is best excited with sounds at its resonance frequency: about 500–750 Hz. For the utricle this is less clear. Its resonance frequency is located at 100 Hz (60), but some reports state that it is most easily excited at higher frequencies around or over 750 Hz (61). An increase in sounds around 500–750 and 750–1,000 Hz in daily life may thus be expected to cause a slight, continuous increase in saccular and utricular activity, respectively. Observing these facts then, this hypothesis predicts that a downward shift of the TMRF, brought about by vTCC activation, will increase sound around 600 Hz, hence excite the saccule and make the individual more perceptive for accelerations in the vertical plane and less aware of linear acceleration in the horizontal plane (Figure 6P). Shifting TMRF upward is then expected to excite utriculus, with the opposite effects. Tympanic dissonance related vestibular symptoms related to inappropriate excitation of utriculus/sacculus are expected to be related to exaggerated/decreased awareness for linear acceleration in the horizontal/vertical plane, and problems with saccadic eye movements: disequilibrium and falling while sitting or standing, but feeling better when lying down; proneness to car sickness; experiencing symptoms related to saccadic eye movements. Or conversely, experiencing disequilibrium when looking down, e.g., during stooping, that decreases when getting upright again; problems related to visual lag, and proneness to sea sickness.

*Sensory symptoms.* The commonest **local sensory symptom**, and the one that most readily responds to patching, is a fullness feeling in the ear (3) (pressure, pain, pangs, feeling of water, plane feeling, …). In dTCC activation accompanied by temporal headache, in vTCC activation radiating toward the hyoid and/or the mastoid. **Referred sensory symptoms** follow the logic of innervation anatomy (Figure 10). In dTCC activation, neuropathic complaints may include burning mouth syndrome, palatal pain, frontal headache, pain over the cheek, nasal bridge or nasal root, tension type headache. In vTCC activation complaints may include ear pain extending below toward the anterior neck or posterosuperior from the ear, at the mastoid level, itching very deep in the ear (“between ear and nose”), and the symptoms of Laryngeal Sensory Neuropathy (6): throat pain, coughing, lump feeling, trouble swallowing, feeling of slime in the throat. In strong sensitization, referred symptoms further away in the body may be possible (tingling feeling in the legs, ischialgia).

*Muscular symptoms.* Muscular symptoms in dTCC activation such as middle ear muscle spasm or tension type headache, relate to masticatory and dorsal neck muscle tensions; and are accompanied by pain and tender points on palpation of these muscles. vTCC activation would then result in palatal muscle spasms, lump feeling, swallowing problems. Increased proprioception causes pain and tender points on palpation of the prevertebral muscles, SCM, trapezius muscle.

*Other symptoms.* This hypothesis predicts that **central symptoms**, such as anxiety, mental stress, fatigue, loss of concentration are not only provoking factors but can be secondary to Tympanic Dissonance. **Tinnitus** covers a broader spectrum, involving central changes, brought about by neural plasticity. While non-disturbing tinnitus as a secondary complaint appears to disappear quite readily after tympanic patching (unpublished data), longstanding and disturbing tinnitus as a primary complaint does only rarely so. Common pathologic middle ear conditions such as otitis media with effusion, ear wax impaction, tubal dysfunction, PET, otosclerosis, … often cause tinnitus that disappears at once or shortly after the condition is treated. In the present hypothesis, these conditions shift TMRF, activate TCC, with subsequent contraction of the related muscles and increased somatosensory input toward the DCN and IC: a known factor in the occurrence or maintenance of somatosensory tinnitus (62). “Middle ear tinnitus” then becomes a specific sub-type in the class of somatosensory tinnitus, which disappears readily when the pathological middle ear condition is treated and the impulses toward the DCN and IC cease, on the condition that no neural plasticity has yet taken place yet.

Of note is that ear symptoms do not necessarily have to be present: many patients with e.g., tension type headache, pharyngeal complaints, … without any ear complaint appear to respond very well to tympanic patching: these may then be caused by Tympanic Dissonance.

#### Relation to Existing Syndromes

Several related existing syndromes form a part of Tympanic Dissonance. In dTCC-related “Tensor tympani syndrome” (5), emphasis is put on TT contraction. When a dental origin of TT contraction is suspected, it is called “Costen syndrome [related: otognatic syndrome, otomandibular syndrome (63–65)]. When TT contraction is compensatory to PR/ET dysfunction, and if some autophony is present, it is commonly called “PET.” In “acoustic shock syndrome” (51), the emphasis rests on sustained listening effort and central auditory effects.

In vTCC-related Laryngeal sensory neuropathy (6), symptoms reflect the activation of the pharyngeal plexus; in this hypothesis caused by increased vTCC activity (related: vagal neuropathy, chronic laryngopharyngeal neuropathy, superior laryngeal nerve syndrome, superior laryngeal neuralgia, hyoid bone syndrome).

#### Diagnostic and Therapeutical Pathway

Tympanic Dissonance should be suspected in patients with unexplained Head & Neck symptoms, accompanied by typical muscle tensions. History taking is very important. The antecedents list provides indications for peripheral or central sensitization: Achilles tendinitis, hip pain, carpal tunnel surgery, … Questionnaires e.g., for anxiety give additional information. Clinical exam includes general appearance, tympanoscopy, endoscopy of the nasopharynx for PR/ET, palpation of the neck and masticatory muscles. Audiometry assesses low frequency hearing loss. Furthermore, a diagnostic consult with the kinesiologist and psychologist. CT for diagnosing third window syndromes and localized inflammation in the mastoid cells. Finally, a short diagnostic trial treatment with anxiolytics is often an eye-opener for both patient and physician. MRI may be useful for neurovascular conflicts (Vth or Xth nerve), cerebral fluid pressure problems, brain stem pathology and to rule out vestibular schwannomas. At the end of the diagnostic workup the dots can be connected and a tentative profile made up for this particular patient: the final profile will only gradually emerge, when first results of therapy provide additional clues.

**Figure 12:**
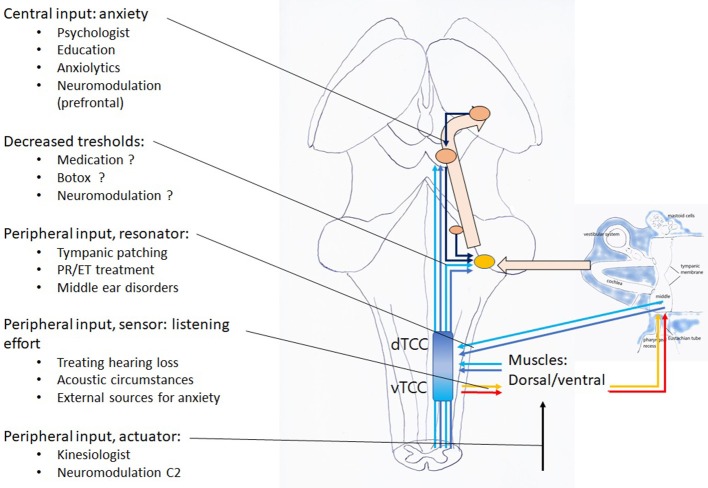
Treatment options.

A treatment based on this hypothesis should address identified inputs (Figure 12). The suggestions for treatment proposed are based on unpublished clinical experience. In this experience, patching appears to provide surprising and far more lasting results than would be suspected from a purely symptomatic therapy. One to three small, slightly wet, rectangular 2–4 mm cigarette paper patches (Rizla blue cigarette paper, 14.5 g/m^2^; Lacroix, Wilrijk, Belgium) are dipped in alcohol 70% and then gently laid onto the tympanic membrane: two superior ones anterior and posterior of the malleus, and a larger inferior one, reniform, covering the lower hemitympanum (Figure 1). The following guidelines have evolved from trial and error: the correct location for patching does not depend on the specific symptom, but on the underlying mechanism. Whenever you see a flaccid area, patch it. If not, patch the ipsilateral upper hemitympanum in dTCC activation and the lower, bilaterally, in vTCC activation (27). The beneficial effect often lasts after dislodgment of the patch, other patients need two or three treatments for a definitive result (1–3). A more lasting result may sometimes be obtained by reinforcing the TM with e.g. a thin slice of cartilage (62). Apart from clicking sounds or some pain on swallowing when patches dry, adverse effects do not occur (1–3). Patching is not recommended in obviously anxious patients, as the nocebo effect may bring these patients to a next level of anxiety with a lasting increase of their initial complaints (e.g., when complaints become temporarily worse, when the wrong location has been patched). In these patients, one may first prescribe anxiolytics, start psychological counseling and/or tDCS of the prefrontal area, and only afterwards patch for the remaining complaints if still needed. Etiological treatment is possible for conditions as inflammation of the mastoid, intracranial pressure, PR/ET inflammation or adhesions. This hypothesis implies that, when proposing surgery (e.g., closing the ET or a SSCD), the relative weight of the targeted factor among the other factors in the whole picture should be established and non-surgical treatment options for every one of these factors discussed, before proceeding to surgery. In the light of the present hypothesis also, decreasing listening effort should be helpful: treating hearing loss, changing acoustic circumstances at the workplace, installing an “acoustic rest” between long stretches of auditory attention. Treat muscular tensions and the underlying causes (physiotherapeutic, orthopedic, dental; addressing posture problems; pilates exercises, …). Sensitization related problems may be treated if possible; and finally address controller problems as anxiety and mental stress, by medical and psychological methods (cognitive therapy, sometimes mindfulness, yoga, …). An interesting and promising evolution is the combination with neuromodulation (at this moment mostly tDCS of the prefrontal and C2 regions) which aims at treating the same phenomena, at a higher central network level and may possibly act by decreasing the central input to both DCN/IC and TCC.

## Discussion

### Arguments in Favor of This Hypothesis

Arguments in favor of this hypothesis relate to the hypothesis itself, the actuators, and the existence of a feedback loop.

#### Embryological and Evolutionary Arguments for the Hypothesis

The spinal nerves deriving from the mantle layer of the neural tube, form dorsal and ventral branches. The dorsal side of the body is concerned with defense against external danger; the ventral side with feeding, communication, care. The hypothesis respects the connection of the dorsal cervical nerves and ventral cervical plexus with this primal function. The muscles innervated by the trigeminal nerve and the facial nerve derive from the first two pharyngeal arches. The derivates of the 3th and 4th pharyngeal arches are often grouped together as glossopharyngeus-vagus-accessorius complex and vagus-accessory complex. Both SCM and trapezius muscles also derive from these lower pharyngeal arches. Again, the hypothesis follows the functional separation, in which the first two pharyngeal arches are concerned with awareness for external stimuli, while the 3th and 4th arch are concerned with feeding, communication, care.

The existence of a peripheral part in the auditory attention network appears logical on evolutionary grounds: analog filtering and pre-processing of data allows for faster central processing, requiring far less computing power. The hypothesis proposes that the tympanic membrane brings interesting auditory information toward the cochlea, in a similar way as extra-ocular and ciliary muscles bring interesting visual information toward the fovea, tongue muscles interesting food to the taste buds on the tongue, and nasal vestibulum muscles interesting smells toward the olfactory nerve.

The peripheral input to the DCN and IC has been suggested to keep the body at standstill and “to suppress responses to ‘expected' body-generated sounds such as vocalization or respiration. This would serve to enhance responses to ‘unexpected' externally-generated sounds, such as the vocalizations of other animals (66).” Contraction of the dorsal cervical muscles when scanning for unexpected stimuli also exposes the face with its antennae (auditory, visual, olfactory, soft touch, …) to the external world, which helps in maximizing external world information gathering. Similarly, in situations of fear, a great many facial muscles cooperate to enhance visual-field size, saccadic velocity, and nasal inspiratory capacity. In situations provoking disgust, sensory exposure is reduced in an antagonistic way by relaxation of these muscles, and contraction of other cervical and facial muscles (48). The muscular mechanism acting on auditory sensory exposure, as proposed here, fits nicely in this general pattern.

The assumption that this pathway, which originates in the peripheral muscles, might finally alter our auditory perception is a step further, but again, similar far-reaching central effects have been found for contraction of other muscle groups (67).

The parallel utricular/saccular and dTCC/dTCC dichotomy is logical from an evolutionary point of view. In dTCC activation, the head is kept upright and horizontal in order to maximally expose the antennae toward the external world. Utricular excitation provides increased awareness for linear acceleration; increased activity of the extra-ocular muscles allows for better scanning for unexpected stimuli. In vTCC activation, the individual bows the neck and turns his attention to the ground for domestic purposes. The head is tilted over 90°: accelerations in the horizontal plane are no longer measured by the utricle, but by the saccule! In this position, better head control is achieved by vTCC related activation of the trapezius and SCM muscles. Not surprisingly, testing of utricular function is done by measuring extra-ocular muscle activity, while SCM activity is measured in saccular function testing.

Moreover, the connection between utricular activation and extra-ocular muscle activity resonates with the well-known cross-modal interactions between oculomotor function and spatial aspects of auditory attention: when listening carefully, one shifts his eyes sideways to the direction of the sound source (when listening even more intently, ipsilateral masticatory, facial and auricular muscles contract as well—dTCC).

#### Clinical Arguments for the Hypothesis

Tympanic patching and other TM stiffness modulating methods proved to be equally effective for autophony (1, 53, 62) and for fullness in the ear (3). The many accompanying symptoms, even if not studied separately, are mentioned in these papers.

The appearance of ear symptoms in seemingly unrelated ailments can only be explained with a common underlying pathophysiological mechanism. Without this hypothesis, one cannot explain why autophony in PET is often accompanied by a fullness feeling in the ear, a slight hearing loss, a slight tinnitus, some slight dizziness; nor can one explain the occurrence of autophony in ailments that are not related to ET opening. Nor the appearance of pulsatile tinnitus in seemingly unrelated ailments; or even tinnitus in middle ear disorders. The referred sensory complaints from muscular tensions described in myofascial pain textbooks often match those of Tympanic Dissonance; a striking example being the description of utriculus/sacculus type equilibrium symptoms, pharyngeal complaints e.g., coughing, and tinnitus-like resonances resulting from SCM tensions (68) that accurately matches the symptoms in vTCC activation. Again, this peculiar combination of symptoms is difficult to explain without the present hypothesis.

#### Technical Arguments Related to Slow and Medium Actuators

The middle ear air cushion effect, including the influence of pars flaccida, has been documented quite extensively (30, 32, 69–72). The effect of mastoid *volume*, in an artificial ear (73) and in cadaveric ears (74) is, as expected, most pronounced for the lower frequencies (“combination of multiple resonators”). The net effect of mastoid *pneumatization* however, where many small air cells are taken into account (“resonator tree”), centers also on the mid-frequencies around 2,000 Hz (75).

#### Technical Arguments Related to Fast Actuators

The effects of TT contraction on TMRF have been well-studied. Moderate TT contraction increases the stiffness of the TOS and shifts the overall RF of the TM upward, damping lower frequencies and promoting mid-range frequencies (23–26). As concerns the (until now unknown) function of TT, the enumeration of seemingly unrelated triggers for TT contraction [after stimulation of certain facial areas (11, 12), on contraction of certain muscles (13, 14), as part of the startle reaction (15, 16), and on speaking or the intention to speak (17), during belching, yawning, and swallowing, but without contributing to ET opening (18)] suddenly makes sense. Indeed TT contraction logically follows, or precedes 1/any act that increases awareness of body sounds, such as speaking, swallowing, masticating, …; and 2/any sign that may signal unexpected danger (light touch for skin and whiskers, stimuli that trigger the startle reaction). The limited sensory innervation and scarcity of muscle spindles found in certain species (76) suggests that the TT muscle, in contrast to other skeletal muscles, does not act on a neuromuscular feedback loop, but on another regulating mechanism, with receptors in the cochlea (74, 77) or in the TM (78).

The notion of fullness feeling in the ear being caused by TT contraction has been around for a long time (32, 71, 72). There are no data whatsoever for the notion of fullness feeling being caused by contraction of PR/ET related muscles. dTCC activation by TT overuse has been postulated in the case of acoustic shock (79). As for the PR/ET complex hypothesis: a more elaborate argumentation can be found in (27).

#### Anatomical Arguments for the Existence of a Feedback System

In contrast to the baroreceptors found on the medial wall of the middle ear, the stretch receptors on the TM are not involved with pressure regulation: impulses resulting from TM vibrations provoke centrally mediated responses related to pharyngeal activity and perhaps TT contraction (80). The Ruffini corpuscles in the nasopharynx were not found on the tubal cartilage or inside the ET but in the PR fundus and posterior nasopharyngeal wall (39), which suggests a function related to the PR/ET complex rather than pressure regulation.

The dTCC/vTCC concept is based on clinical experience with tympanic patching, where two distinct patient groups emerge. The exact anatomic pathway from the vagal nerve to the TCC is not clear: is it a direct connection to the spinal trigeminal nucleus, or an indirect one via the solitary tract nucleus? Transcutaneous vagal nerve stimulation, via the auricular branch of the vagal nerve, induces FOS immunoreactivity (an indirect marker for neuronal activity) in the solitary tract nucleus and trigeminal nuclei (81); stimulation from the antero-lateral aspect of the neck activates solitary tract nucleus but inhibits spinal trigeminal nucleus (82). These observations favor an indirect way, in which vagal stimulation could increases activity at the solitary tract nucleus level, which might then inhibit activity in the trigeminal nuclei. On the other hand, the auricular branch of the vagal nerve is known to also connect directly with the trigeminal nuclei (40, 41) and a direct vagal activation of the trigeminal nuclei has been suggested (83). The term “trigeminovagal complex” (84), coined for this direct connection, may therefore depict the same functional unit as the proposed “vTCC.”

There are some problems with the nomenclature, related with the term “trigeminal” designating at once the brain stem nuclei and the peripheral nerve. The term “trigeminocervical complex” designates, at the brain stem level, a complementary link between the cervical nuclei and trigeminal nuclei; clinically, this translates in a complementary function of peripheral cervical and trigeminal nerves. The term “trigeminovagal” suggests a complementary link between vagal and trigeminal nuclei; this term, clinically used, would seem to imply a complementary functioning of the peripheral vagal and trigeminal nerves. In the present hypothesis however, the input of trigeminal and vagal nerve is seen as antagonistic. Mirroring the complementary input of dorsal cervical and trigeminal nerves, a complementary input between vagal nerve and cervical plexus is assumed. The terms “trigeminocervical and vagocervical complex” (27), are intuitive from a clinical point of view (as they designate peripheral structures), but perhaps less accurate and systematic for scientific purposes. Until more is known about the exact mechanisms involved and the value of the statements proposed here, the terms dTCC and vTCC may be a prudent choice in the context of this hypothesis.

#### Functional Arguments for the Existence of a Feedback System and the Concept of Resonance Homeostasis

Insight in the role of compensation mechanisms can be gained by comparing clinical observations and experiments in live ears, and experimental settings using artificial or cadaveric ears, where the compensation mechanism is abolished.

*In live ears*, the stiffness of the TOS has been measured using various techniques. In fenestral otosclerosis, calcification of the annular ligament around the stapes footplate (at the OW membrane) is expected to increase TOS stiffness. However, this upward shift appears to be much less pronounced than the one found in e.g., malleus fixation, a non-hereditary disease (85–88); there is significant overlap with normal ears, and sometimes even a downward shift (89)!

In this hereditary disease, slowly acting mechanisms play a significant role. For the early otologists, who were often confronted with longstanding and advanced cases of fenestral otosclerosis, inspection of PR/ET offered the principal diagnostic clue in distinguishing otosclerosis from disease secondary to inflammation or tympanosclerosis (90)! Sourdille (91) describes a very hard, large and long, almost ossified tubal cartilage with a wide open PR in otosclerosis, as opposed to inflammation in the PR in inflammatory disease. He also mentions a larger TM size (larger TM: lower TMRF). Recently, increased pneumatization has been documented in the mastoid in otosclerotic patients (92, 93): another slow acting mechanism.

The effect of cochlear load on TOS RF has been measured *under experimental conditions*: when no compensation mechanism is present, intracranial hypertension as well as hypotension both produce an upward shifting effect (94, 95). In third window syndromes as SSCD, there is a local dehiscence in the thick and hard bone that normally surrounds the cochlea. When artificially produced *in cadaveric ears*, such dehiscences cause a downward shift of TOS RF (96). In patients suffering from a *symptomatic* third window syndrome (87), a similar effect was seen, but the effect was not present in all patients; again, this suggests the presence of a compensation mechanism. SSCD patients have been living with an anatomical dehiscence from childhood, but only develop symptoms at a specific moment in adulthood. This is only possible if a combination of several factors account for the symptoms. *Symptomatic* SSCD patients (89) do indeed have smaller mastoids (less possibility for compensation—more chance of developing symptoms). *Symptomatic* PET patients also have been found to have smaller mastoids (81, 97), and PET patients with only unilateral symptoms (98) often have a bilateral open ET. Again, this means that, with correct compensation mechanisms, a wide open ET does not need to produce autophony. Indeed, a flaccid area, moving in and out with breathing, can often be noticed in asymptomatic patients. Similarly, in pulsatile tinnitus, a dehiscence of the jugular bulb or sigmoid sinus have been present since birth while complaints only arise during adulthood.

Patients with symptomatic PET often have a sniffing habit, which induces “a better feeling in the ear” with less fullness feeling. This compensatory habit aims at shifting TMRF upward by building a negative middle ear pressure. In most cases the habit disappears after tympanic patching.

### Arguments Against the Hypothesis

The hypothesis fails to explain why many patients with third window syndrome or intracranial hypotension do not feel any fullness feeling. Perhaps, at the time of diagnosis, they rely solely on slow or very slow acting mechanisms. It fails to explain why patients do not develop Tympanic Dissonance symptoms after middle ear surgery, when gross changes are made to the mastoid and TM. Thickening of the TM may damp the whole system, nerves may be sectioned, and hearing loss may mask symptoms. Some patients however do complain of unexplainable dullness of hearing after uneventful surgery, but in the absence of adequate testing methods these observations are mostly dismissed.

What about TT sectioning e.g., in Meniere's disease? The hypothesis predicts that these patients will afterwards use the remaining masticatory muscles in the PR/ET system for upward shifting, and this appears to be the case indeed (16).

Birds and reptiles possess a columella without muscles, and cannot make use of this system; these animals have an extended pneumatization in the skull. This pneumatization, generally considered to be an evolutionary adaptation to save weight, was present in the large dinosaurs, who possessed a columella as well and in whom weight saving was not important. Moreover, in birds and other species the ET's from both sides fuse before they connect to the nasopharynx. The resulting acoustic coupling of the ears offers in itself a very effective tool for focusing and sound localization (99). One may speculate that TRRS in mammals evolved when both ET's became separated.

Attempts to measure TMRF changes related to listening have proven unsuccessful until now. Measurements need to be very precise, and a more sophisticated setup in a specialized lab may be able to find the hypothesized effect.

It is unclear how changes in TMRF in frequency domain, shifting upward and downward, are related to attention to unpredictable and predictable events. Body sounds are predictable; external sounds can be predictable or unpredictable. As TT muscle contraction on speaking or the intention to speak damps the lower frequencies, one would expect predictable (body) sounds to be linked with low frequencies, and unpredictable stimuli with mid-frequencies. However, the concept of “low vs. mid-frequencies” may be too simplistic, and more complex mechanisms may be at play. Obstacles to test this hypothesis are foremost related to the diversity of the symptomatology and interference with psychological factors; the compensatory mechanisms at play; and the lack of a measurement system that allows for objective measures. Clinical studies, in the form of rigorous single-blind or double-blind studies are therefore restricted to studying only one or at most a few of the symptoms at a time. These are not likely to provide fundamental new insights in this multi-faceted pathology. As for now, these are based on questionnaires and psycho-acoustic tests. An objective measuring method should necessarily measure the response of this complex system to a standardizes stimulus. VEMP testing is such a method, and measures one pathway involved in TRRS. Stimulating the TRRS e.g., by electric stimulation of the tongue (12), combined with reflectance measurements of the TM and sonotubometry might be a possibility. Operative TT sectioning, in humans or animals, combined with reflectance measurements, sonotubometry, EMG in order to detect whether the subject tends to more intensely use the PR/ET system after TT sectioning. Anatomically characterizing the nerve receptors on the TM and PR, the nerves they are connected to, their connections to central structures.

### Place of the Hypothesis in Context of Current Views

On a clinical level, this hypothesis brings together complaints and syndromes that until now seemed unrelated, “vague,” and difficult to diagnose and treat. This framework offers possibilities for development of diagnostic and therapeutic measures. It offers the field of otology, until now rather focused on the ear as a separate entity, a vision more integrated with general medicine and psychology.

Long ago, the concept of TCC has been deduced from the clinical observations of co-occurrence of cervical and masticatory muscle tensions, stress and anxiety, and symptoms around the ear. This hypothesis provides an explanation for these observations in attributing a function for TCC as a pivotal part of the brainstem pathways involved in auditory attention.

It also attributes a function to the Tensor Tympani muscle and PR/ET complex and gives new insights in the role of middle ear structures. It explains many observations in otology such as the connection between mastoid pneumatization and diseases; or why long standing anatomic defects such as SSCD only cause symptoms in adulthood, why many ear symptoms are linked to psychological moods, etc. It offers new therapeutic possibilities for these middle ear problems.

The system concerned with auditory attention has, until now, been considered as being organized on a purely central level. By adding a link between muscles, central elements and tympanic membrane, the hypothesis firmly extends this peripheral part related with auditory attention (Figure 11), so that it becomes a fully integrated system, allowing for diagnostic and therapeutic measures.

## Conclusion

The concept of Tympanic Resonance provides a unifying hypothesis, that allows to explain the pathophysiology of a wide array of symptoms that are encountered extremely frequently in clinical practice, in ailments that until now seemed unrelated. It provides a connecting link between several existing but poorly defined syndromes; allows for new insights on the function of certain elements of the middle ear, the trigeminal and vagal nerve, and a more “integrative” view on ear pathology.

It adds a peripheral part to the complex system that is concerned with auditory attention and ultimately vigilance. It provides a “raison d'être” for the well-known concept of TCC, as a pivotal brainstem integration center in the pathway involved in the modulating and directing of auditory attention, and the transformation of auditory sensation to perception.

Future studies to underpin the various concepts in this hypothesis are welcomed.

“A wing would be a most mystifying structure if one did not know that birds flew” (100).

## Author Contributions

The author confirms being the sole contributor of this work and has approved it for publication.

### Conflict of Interest

The author declares that the research was conducted in the absence of any commercial or financial relationships that could be construed as a potential conflict of interest.

## Abbreviations

TM: Tympanic Membrane
TOS: Tympano-ossicular System
OW: Oval Window
RW: Round Window
TT: Tensor Tympani muscle
ET: Eustachian Tube
PR: Pharyngeal Recess
TMRF: Tympanic Membrane Resonance Frequencies
RF: Resonance Frequency
VCN: Ventral Cochlear Nucleus
DCN: Dorsal Cochlear Nucleus
IC: Inferior Colliculus
PET: Patulous Eustachian Tube Syndrome
TRRS: Tympanic Resonance Regulating System
TCC: Trigeminocervical Complex
dTCC: Dorsal (or trigeminal) Trigeminocervical Complex
vTCC: Ventral (or vagal) Trigeminocervical complex
SCM: Sternocleidomastoid Muscle
SSCD: Superior Semi-circular Canal Dehiscence.
